# Cell-bound complement activation products in antiphospholipid antibody-positive patients without other systemic autoimmune rheumatic diseases

**DOI:** 10.3389/fimmu.2024.1459842

**Published:** 2024-09-17

**Authors:** Doruk Erkan, Joann Vega, Tyler O’Malley, Andrew Concoff

**Affiliations:** ^1^ Barbara Volcker Center for Women and Rheumatic Diseases, Hospital for Special Surgery, New York, NY, United States; ^2^ Department of Medicine, Weill Cornell Medicine, New York, NY, United States; ^3^ Clinical Affairs, Exagen, Vista, CA, United States

**Keywords:** antiphospholipid syndrome, antiphospholipid antibodies, complement activation, cell-bound complement activation products (CB-CAPs), disease activity

## Abstract

The objective of this study was to analyze complement activation in antiphospholipid antibody (aPL)-positive patients without other systemic autoimmune rheumatic diseases, using C3/C4 and cell-bound complement activation products (CB-CAPs) (B-lymphocytes [BC4d], erythrocytes [EC4d], and platelets [PC4d]). Persistently aPL-positive patients with or without aPL-related clinical manifestations (thrombotic APS [TAPS], microvascular APS [MAPS], obstetric APS, thrombocytopenia [TP], and/or hemolytic anemia [HA]) were enrolled in a single center study. Blood and clinical data were collected at baseline; a subgroup of patients completed 6- or 12-month follow-up. At baseline, 4/31 (13%) patients had decreased C3/C4, while 7/29 (24%) had elevated BC4d, 11/33 (33%) EC4d, and 12/32 (38%) PC4d. Based on different aPL profiles, all patients with decreased C3/C4 or elevated BC4d, EC4d, and PC4d had triple aPL or isolated lupus anticoagulant positivity. Based on different aPL clinical phenotypes, the number of patients with strongly positive EC4d and PC4d were proportionally higher in those with MAPS/TP/HA, compared to TAPS or no APS. Compared to baseline, the frequencies of BC4d, EC4d, and PC4d positivity were not significantly different in the subgroup of patients during their 6- or 12-month follow-up. There was a weak correlation between C3/C4 and CB-CAPs, especially for PC4d. In summary, complement activation in aPL-positive patients varies based on aPL profiles and clinical phenotypes. Given the higher percentage of aPL-positive patients with abnormal CB-CAPs, compared to C3/C4, and the poor inverse correlation between CB-CAPs and C3/C4, our study generates the hypothesis that CB-CAPs have a role in assessing disease activity and thrombosis risk in aPL-positive patients.

## Introduction

Antiphospholipid syndrome (APS) is a heterogeneous autoimmune disease with thrombotic, obstetric, microvascular, and non-thrombotic manifestations, which may coexist with other systemic autoimmune rheumatic diseases (SARDs), especially systemic lupus erythematosus (SLE) (1). Antiphospholipid syndrome can also develop without other SARDs (primary APS). In antiphospholipid antibody (aPL)-positive patients, the risk of first or recurrent thrombosis increases with high-risk aPL profile, e.g., triple aPL-positivity, and with additional venous thromboembolism and cardiovascular disease risk factors; however, there are no biomarkers to predict future thrombosis. Similarly, patients with microvascular APS, e.g., diffuse alveolar hemorrhage, and non-thrombotic manifestations, e.g., thrombocytopenia (TP) generally have ongoing disease activity with no biomarkers to document or monitor disease status.

Cell-bound complement activation products (CB-CAPs) are complement-split products covalently bound to blood cells. Because they are formed upon classical complement activation, complement protein 4 degradation (C4d) accumulation on hematopoietic cells reflects complement system dysregulation. Covalent bonding imparts stability to the complement signature represented by CB-CAPs in contrast to soluble complement fragments vacillating between various stages of equilibrium (2).

Based on animal models of thrombosis and pregnancy morbidity, complement activation is part of APS pathogenesis (3). However, studies investigating complement activation in aPL-positive patients, especially based on different aPL profiles and clinical phenotypes are limited. Thus, our primary objective was to analyze complement activation in different subgroups of aPL-positive patients, using complement protein 3/4 (C3, C4) and CB-CAPs (B-lymphocytes [BC4d], erythrocytes [EC4d], and platelets [PC4d]).

## Methods

In this longitudinal prospective single-center pilot study, persistently aPL-positive (≥ 12 weeks apart; last aPL positivity within six months (m) prior to entry) adult patients without other SARDs were enrolled. Positive aPL was defined as positive lupus anticoagulant (LA) test, anticardiolipin antibody (aCL) IgG/M ≥ 40 enzyme-linked immunosorbent assay (ELISA) units, and/or anti-β_2_-Glycoprotein-I antibody (aβ_2_GPI) IgG/M ≥ 40 ELISA units. For those with aPL-related manifestations at least one event within the five years prior to enrollment was required. Selected exclusion criteria were active infection, pregnancy, cancer, and corticosteroid use (≥20 mg prednisone or equivalent per day).

Clinical data (demographics, APS-related medical history and laboratory tests, and medications) and blood samples were collected at baseline for all patients; a subgroup of patients completed 6m (+/- 1m) or 12m (+/- 1m) follow-up for additional clinical data and blood collection.

For the purpose of group comparisons, patients were grouped first based on their aPL profiles and then aPL-related clinical phenotypes. Antiphospholipid antibody profiles were defined as: a) triple aPL-positivity (positive LA, aCL IgG/M and aβ_2_GPI IgG/M); b) single LA or double aPL (including LA) positivity; c) double aPL (excluding LA) positivity (aCL IgG/M and aβ_2_GPI IgG/M); and d) single aPL (excluding LA) positivity (aCL IgG/M or aβ_2_GPI IgG/M). Antiphospholipid antibody-related clinical phenotypes were defined as: a) microvascular APS (MAPS) (i.e., diffuse alveolar hemorrhage, aPL-nephropathy, cardiac microthrombosis, and/or livedoid vasculopathy) and/or non-thrombotic APS (i.e., autoimmune TP [platelet count persistently <150x10^9^/L] and/or autoimmune hemolytic anemia [HA] with hemolysis and with a positive direct antiglobulin test) with or without thrombotic APS (TAPS) or obstetric APS (OAPS); b) TAPS with or without OAPS based on the revised Sapporo APS classification criteria (4); and c) asymptomatic aPL-positivity (although the original protocol included the analysis of patients with only OAPS, we were not able to recruit patients for this group).

Blood samples were measured for anti-phosphatidylserine-dependent prothrombin antibody (aPS/PT), C3/C4, BC4d, EC4d, and PC4d. Anti-phosphatidylserine-dependent prothrombin antibody was measured by ELISA; anti-PS/PT IgG values > 37 Units and anti-PS/PT IgM values > 30 Units were considered positive. Complement 3/4 were measured by turbidimetry (5); low C3 was defined as levels below 81mg/dl, and C4 as below 13 mg/dl. B-lymphocyte C4d, EC4d, and PC4d were measured in ethylenediaminetetraacetic acid (EDTA) anticoagulated blood by semiquantitative flow cytometry at Exagen as described previously (2). Briefly, cells were stained with a mouse monoclonal antibody against C4d or a non-specific isotype control antibody and with a fluorescent secondary antibody. The cells were analyzed by flow cytometry and the mean fluorescence intensity (MFI) of the isotype control (background) is subtracted from the C4d-specific antibody fluorescence intensity to determine the net MFI. Cutoff values for BC4d, EC4d, and PC4d positivity, based on the 99^th^ percentile of a group of healthy individuals, were 60, 14, and 9 net MFI, respectively; however, data analysis was performed based on “positive” (99^th^ percentile of healthy population) and “strong positive” (99^th^ percentile of SARD population) results (**Table 1** Footnote).

**Table 1:** Complement activation markers in persistently antiphospholipid antibody (aPL) positive patients without other systemic autoimmune rheumatic diseases, baseline results overall and by aPL profile.

#abnormal/# tested or Mean (SD)	Total (n: 33)	Triple aPL (LA, aCL & aB_2_GPI) (n:21)*	Single LA or Double aPL (LA & aCL or aB_2_GPI) (n:5)**	Double aPL (aCL & aB_2_GPI) (n: 5)	Single aPL (aB_2_GPI) (n:2)
C3 (mg/dl)					
<81	4/31 (13%)	3/19 (16%)	1/5 (20%)	0	0
*Mean*	113.6 (32.1)	105.4 (35.0)	129.3 (38.6)	114.3 (20.3)	126.0 (34.8)
C4 (mg/dl)					
<13	4/31 (13%)	3/19 (16%)	1/5 (20%)	0	0
*Mean*	25.2 (8.8)	22.7 (10.0)	27.3 (6.2)	25.5 (7.8)	34.5 (0.4)
BC4d Level (MFI)					
61-100 ^a^	1/29 (3%)	1/19 (5%)	0	0	0
101-200 ^b^	6/29 (21%)	6/19 (32%)	0	0	0
>200	0	0	0	0	0
*Any Positive*	*7/29 (24%)*	*7/19 (37%)*	*0*	*0*	*0*
*Mean*	*47.5 (44.8)*	*61.0 (52.0)*	*28.0 (17.8)*	*28.0 (17.4)*	*17.5 (4.9)*
EC4d Level (MFI)					
15-30 ^c,d^	7/33 (21%)	7/21 (33%)	0	0	0
31-75 MFI ^e^	2/33 (6%)	2/21 (10%)	0	0	0
>75 MFI ^f^	2/33 (6%)	2/21 (10%)	0	0	0
*Any Positive*	*11/33 (33%)*	*11/21 (52%)*	*0*	*0*	*0*
*Mean*	*18.2 (25.4)*	*25.7 (30.3)*	*8.6 (3.8)*	*4.9 (0.8)*	*4.8 (1.8)*
PC4d Level (MFI)					
10-15 MFI ^g^	2/32 (6%)	1/21 (5%)	1/4 (25%)	0	0
16-20 MFI	1/32 (3%)	1/21 (5%)	0	0	0
>20 MFI ^h^	9/32 (28%)	7/21 (33%)	2/4 (50%)	0	0
*Any Positive*	12/32 (38%)	9/21 (43%)	3/4 (75%)	0	0
*Mean*	38.3 (104.6)	26.8 (35.1)	154.9 (282.8)***	5.1 (2.0)	3.0 (0)
Any Positive CB-CAP^*,**^.	16/33 (49%)	13/21 (62%)	3/5 (60%)	0	0

Positive as 61-200 mean fluorescent intensity (MFI), 15-75 MFI, and 10-20 MFI, and Strongly Positive above 200 MFI, 75 MFI, and 20 MFI for BC4d, EC4d, and PC4d, respectively. LA, lupus anticoagulant; aCL, anticardiolipin antibody; aβ_2_GPI, anti-β_2_-Glycoprotein-I antibody. *Two patients with no C3/C4 and BC4d results. ** Two patients with no BC4d results, and one with no PC4d result. ***Based on four samples with levels of 6 MFI, 11.5 MFI, 23 MFI, and an outlier of 579 MFI. ^a^ Six month (m) repeat BC4d was in the same range (SR) (1 of 1 tested); ^b^6m BC4d SR (2/2); ^c^6m EC4d SR (3/4) and 31-75 range (1/4); ^d^12m EC4d SR (1/3), negative (1/3), and 31-75 range (1/3); ^e^6m EC4d SR (2/2); ^f^6m EC4d SR (1/1); ^g^6m PC4d negative (2/2), and 12m PC4d SR (1/2) and negative (1/2); and ^h^6m PC4d SR (3/5), 10-15 range (1/5), and 16-20 range (1/5), and 12m PC4d SR (1/1).

Following a descriptive analysis of the baseline C3/C4 and CB-CAP results based on different aPL profiles and clinical phenotypes (frequencies and means as appropriate), baseline C3/C4 and CB-CAP levels were correlated (Spearman’s p correlation test). As a subgroup analysis, in patients with 6m or 12m blood collection, we also analyzed the persistency of abnormal CB-CAP results, and compared the proportion of persistent CB-CAP positive patients to baseline results (Fischer’s exact test).

## Results

Between August 2020 and November 2022, 33 patients (female 23 [70%], mean age 50.6 ± 12.5, White 30 [91%]) were enrolled (**Supplementary Table 1**). Antiphospholipid antibody profile of patients is shown in **Table 1** (triple aPL positivity: 21 [64%], single LA or double aPL [with LA]: 5 [15%], double aPL [without LA]: 5 [15%], and single aPL [without LA]: 2 [6%]); 95% of triple aPL-positive patients and 60% of double aPL (with LA) positive patients were also positive for aPS/PT (**Supplementary Table 2**). Antiphospholipid antibody clinical phenotypes are shown in **Table 2**, which were based on not mutually exclusive aPL-related clinical events in 26 patients (79%) (TAPS: 25 [75%], MAPS: 10 [30%], TP: 8 [24%], OAPS: 5 [15%], and HA: 2 [6%]) as well as no aPL-related clinical events in seven patients (21%).

**Table 2:** Complement activation markers in persistently antiphospholipid antibody (aPL) positive patients without other systemic autoimmune rheumatic diseases, baseline results overall and by clinical phenotype.

#abnormal/# tested or Mean (SD)	Total (n: 33)	MAPS/TP/HA* (n:14)**	TAPS*** (n: 12)****	No APS (n: 7)
C3 (mg/dl)				
<81	4/31 (13%)	2/12 (17%)	1/12 (8%)	1/7 (14%)
*Mean*	113.6 (32.1)	117.1 (31.6)	123.4 (34.9)	97.1 (27.8)
C4 (mg/dl)				
<13	4/31 (13%)	2/12 (17%)	1/12 (8%)	1/7 (14%)
*Mean*	25.2 (8.8)	27.7 (8.8)	28.5 (8.6)	17.7 (4.3)
BC4d Level (MFI)				
61-100	1/29 (3%)	1/12 (8%)	0	0
101-200	6/29 (21%)	4/12 (33%)	1/10 (10%)	1/7 (14%)
>200	0	0	0	0
*Any Positive*	*7/29 (24%)*	*5/12 (42%)*	*1/10 (10%)*	*1/7 (14%)*
*Mean*	*47.5 (44.8)*	*48.6 (45.1)*	*42.8 (49.5)*	*53.5 (43.3)*
EC4d Level (MFI)				
15-30	7/33 (21%)	2/14 (14%)	2/12 (17%)	3/7 (43%)
31-75 MFI	2/33 (6%)	1/14 (7%)	1/12 (8%)	0
>75 MFI	2/33 (6%)	2/14 (14%)	0	0
*Any Positive*	*11/33 (33%)*	*5/14 (36%)*	*3/12 (25%)*	*3/7 (43%)*
*Mean*	*18.2 (25.4)*	*27.1 (36.4)*	*12.9 (11.7)*	*9.8 (5.7)*
PC4d Level (MFI)				
10-15 MFI	2/32 (6%)	0	2/11 (18%)	0
16-20 MFI	1/32 (3%).	0	0	1/7 (14%)
>20 MFI	9/32 (28%)	5/14 (36%)	2/11 (18%)	2/7 (29%)
*Any Positive*	*12/32 (38%)*	*5/14 (36%)*	*4/11 (36%)*	*3/7 (43%)*
*Mean*	*38.3 (104.6)*	*62.2 (151.6)*	*23.4 (37.2)*	*9.8 (8.5)*
Any Positive CB-CAP^**,****^	16/33 (49%)	8/14 (57%)	5/12 (42%)	3/7 (43%)

Positive as 61-200 mean fluorescent intensity (MFI), 15-75 MFI, and 10-20 MFI, and Strongly Positive above 200 MFI, 75 MFI, and 20 MFI for BC4d, EC4d, and PC4d, respectively. *Includes (not mutually exclusive) Microvascular APS (lung [active: 1, inactive: 3]; kidney [active: 2, inactive: 1, suspected with abnormal kidney function: 3]; and skin [inactive 1]), autoimmune thrombocytopenia [baseline 100-149 x10^9^/L: 5; inactive: 3), and/or autoimmune hemolytic anemia (active: 1, inactive: 1). 13/14 also had Thrombotic APS and 1/14 also had Obstetric APS (OAPS). ** Two patients with no C3/C4 and BC4d results. ***4/12 also had OAPS. ****Two patients with no BC4d and one with no PC4d results.

Four of 31 (13%) aPL-positive patients had decreased C3/C4, while 7/29 (24%) had elevated BC4d (no strong positivity [SP]), 11/33 (33%) EC4d (6% SP), and 12/32 (38%) PC4d (28% SP) at baseline; the number of patients with any CB-CAP (BC4d, EC4d, and/or PC4d) positivity was 16/33 (49%). Based on different aPL profiles, all patients with decreased C3/C4 or elevated BC4d, EC4d, and PC4d had triple aPL positivity, or LA positivity with/without aCL or aβ_2_GPI (additionally, higher mean EC4d and PC4d levels were observed in these groups) (**Table 1**). Based on different aPL clinical phenotypes, the number of patients with strongly positive EC4d and PC4d were proportionally higher (14% and 36%) in those with MAPS/TP/HA, compared to those with TAPS (0 and 18%) or no APS (0 and 29%) (additionally higher mean EC4d and PC4d levels were observed in the former group) (**Table 2**).

In the subgroup analysis of 20/33 (61%) patients with 6m (n: 20) or 12m (n: 9) follow-up data: a) 3/7 BC4d-positive patients at baseline had 6m repeat tests (all positive); b) 7/11 EC4d-positive had 6m tests (all positive) and 3/11 had 12m tests (2 positive and 1 negative); and c) 7/12 PC4d-positive had 6m tests (5 positive and 2 negative) and 3/12 had 12m tests (2 positive and 1 negative). The proportions of persistent CB-CAP positive patients were 16% (3/19), 30% (6/20), and 30% (6/20) for BC4d, EC4d, and PC4d, respectively, which were not statistically different compared to baseline results (24%, 33%, and 38%) (p: 0.72, 1.00, and 0.58, respectively), although the proportions were numerically lower (**Table 1** Footnote, **Supplementary Tables 3**, **4**, and **Supplementary Figure 1**).

**Figure 1:**
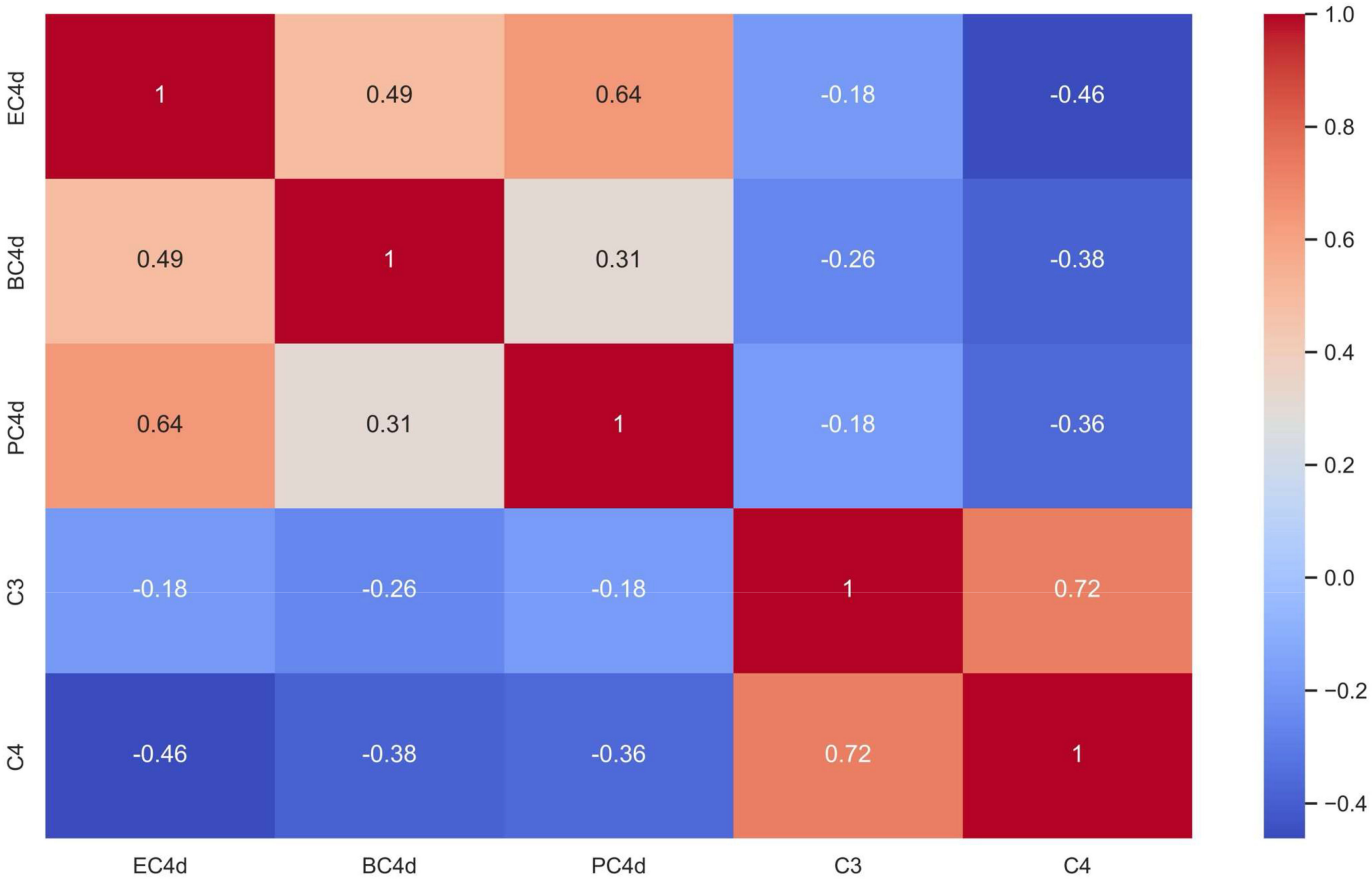
Cell-bound Complement Activation Products (B-lymphocytes [BC4d], erythrocytes [EC4d], and platelets [PC4d]) Correlated to Serum Complement C3 and C4 Levels (Spearman’s rank correlation coefficient [r] is shown with darker red shades representing strong positive correlations and darker blue shades representing strong inverse correlations between biomarkers).

Based on the analysis of all simultaneously tested samples (n: 38), there was a weak inverse correlation between C3/C4 and CB-CAPs, especially for PC4d (r = -0.18 and -0.36 for C3 and C4, respectively) (**Figure 1**).

## Discussion

Based on our small pilot study, cross-sectional assessment of complement activation in persistently aPL-positive patients without other SARDs demonstrated that 13%, 24%, 33%, and 38% had abnormal baseline C3/C4, BC4d, EC4d, and PC4d, respectively; all patients with abnormal results had triple aPL-positivity, or LA-positivity with/without aCL or aβ_2_GPI. The number of patients with strongly positive EC4d and PC4d were proportionally higher (14% and 36%) in those with MAPS, TP, and/or HA. Compared to baseline, the proportions of persistent BC4d-, EC4d-, and PC4d- positive patients were not significantly different in the subgroup of patients with 6- or 12-month follow-up.

Animal studies demonstrated that aβ_2_GPI are associated with complement activation; the anaphylatoxins C5a and C3a may induce procoagulant activity, inhibit fibrinolysis, and activate platelets and endothelial cells with resulting expression of adhesion molecules (3, 6). Complement 3 and C5 activation are also implicated in pregnancy loss, as demonstrated in animal models, mice deficient in C3 or C5 or treated with anti-C5a monoclonal antibodies are protected against pregnancy loss and growth retardation induced by aPL (6, 7). Given that hypocomplementemia (low C3/C4) are detected in selected APS patients (8), and limited data exist investigating complement activation or CP-CAPs in APS, our study is timely to further investigate complement activation as a marker of disease activity and a risk assessment tool in different subgroups of aPL-positive patients.

Complement activation can be evaluated by: a) fluid phase complement assays (C3, C4, complement activation fragments [C3a, C5a, C3d, Ba, Bb], or complement related autoantibodies [anti-C1q, anti-Factor H]); and b) cell-based complement assays (CB-CAPs, hemolytic assays, or functional assays [complement-mediated cell killing – modified HAM test) (6). There have been inconsistent associations between fluid phase complement assays and aPL-manifestations, possibly due to the fact that rather than complement activation, complement deposition on cell surfaces resulting in endothelial injury is more important in APS (6). Our study also demonstrated a higher percentage of persistently aPL-positive patients had any abnormal CB-CAP results (49%), compared to abnormal C3/C4 levels (13%), with a weak inverse correlation between CB-CAPs and C3/C4.

Lonati et al. demonstrated that patients with primary APS, APS-associated with other SARDs, and aPL-positive SLE (without APS) had a higher percentage EC4d- and PC4d-positive cells compared to normal healthy individuals, aPL-negative patients with thrombosis, and patients with idiopathic thrombocytopenic purpura. The percentage of cells positive for EC4d and PC4d was intermediate in aPL-negative SLE patients and asymptomatic healthy aPL carriers. Based on additional *in vitro* experiments, authors also demonstrated CB-CAPs deposition on activated platelets (9). Our study is the first analyzing CB-CAPs based on different aPL profiles and clinical phenotypes, and suggests that: a) not every aPL profile results in complement activation in aPL-positive patients; and b) patients with microvascular disease and non-thrombotic manifestations more commonly have complement activation.

Platelet-bound C4d and, to a lesser extent, EC4d, are associated with a history of arterial and venous thrombosis in SLE (10). Preliminary data show that persistent PC4d positivity during a one-year follow-up of SLE patients is more strongly associated with a history of thrombosis, compared to intermittent PC4d positivity. A “thrombotic composite score” consisting of PC4d, low C3, and LA is higher in SLE patients with history of thrombosis than in patients without this history (10). In our cohort, approximately one-third of APS patients with thrombosis (n:25) were positive for PC4d, compared to 43% of aPL-positive patients with no aPL-related symptoms (n:7). Although the PC4d association with aPL-positivity in SLE patients (11) may explain these similar findings in our two groups of persistently aPL-positive patients, given the small numbers and imbalanced groups, caution is needed when interpreting these findings. Thus, determining the role of PC4d for thrombosis prediction in aPL-positive patients without SLE requires further larger-scale studies.

Our study has several limitations. Firstly, despite the prospective nature of the study, our cohort is relatively small as the patient recruitment was limited and a significant portion of patients could not have follow-up visits due to COVID-related restrictions at the time of the study. Secondly, given the lack of a standardized APS disease activity assessment tool, there may be substantial variability in how disease criteria were observed and documented. Nonetheless, this study provides a number of future research areas for further pursuit.

In conclusion, our study demonstrates that complement activation in aPL-positive patients varies based on aPL profiles and clinical phenotypes. Given the higher percentage of aPL-positive patients with abnormal CB-CAPs, compared to C3/C4, and the poor correlation between CB-CAPs and C3/C4, our study generates the hypothesis that CB-CAPs have a role in assessing disease activity and thrombosis risk independent of serum complement levels in aPL-positive patients. Furthermore, clinical studies are needed to determine if CB-CAPs can be a useful biomarker for clinicians to guide which APS patients might benefit most from complement inhibitors.

## Data Availability

The raw data supporting the conclusions of this article will be made available by the corresponding author upon reasonable request.
