# Supplementary material for: Cell-bound complement activation products in antiphospholipid antibody-positive patients without other systemic autoimmune rheumatic diseases
Source: Front Immunol. 2024 Sep 17;15:1459842. doi: 10.3389/fimmu.2024.1459842 (PMC11443598; doi:10.3389/fimmu.2024.1459842)
Supplement: Supplementary file 1 [file DataSheet1.pdf]

Erkan *et al.*
Cell-bound Complement Activation Products in Antiphospholipid Antibody-positive Patients without Other Systemic Autoimmune Rheumatic Diseases
Supplement

Supplement Table 1: Baseline Demographics, Clinical and Laboratory Characteristics, and Selected Medications of Persistently Antiphospholipid Antibody-positive Patients (n: 33)

**Demographics:**	
- Mean Age at Study Entry (mean $\pm$ SD)	50.6 $\pm$ 12.5
- Female	23 (70%)
- White	30 (91%)
**Clinical Characteristics:**	
- Thrombosis	25 (75%)
- Microvascular Disease[1]	10 (30%)
- Obstetric APS[2]	5/23 (22%)
- Thrombocytopenia	8 (24%)
- Autoimmune Hemolytic Anemia	2 (6%)
- Cardiac Valve Disease	1 (3%)
- No aPL-related Events	7 (21%)
**Laboratory Characteristics:**	
- Triple aPL-positivity	21 (64%)
- Single LA or Double aPL-positivity [with LA]	5 (15%)
- Double aPL-positivity [without LA]	5 (15%)
- Single aPL-positivity [without LA]	2 (6%)
**Disease Duration:**	
- From APS Classification to Recruitment (mean $\pm$ SD, years)[3]	10.3 $\pm$ 4.6
- From the 1st aPL Detection to Recruitment (mean $\pm$ SD, years)[4]	10.7 $\pm$ 8.8
**Medications:**	
- Anticoagulation[5]	23 (70%)
- Low-dose Aspirin	18 (55%)
- Hydroxychloroquine (HCQ)	14 (42%)
- Immunosuppression Other Than HCQ[6]	9 (27%)
- Statins	8 (24%)
- Corticosteroids ($\leq$ 20mg/day prednisone or equivalent)	3 (9%)

APS: antiphospholipid syndrome; **LA**: lupus anticoagulant. [1]Microvascular disease (lung [active: 1, inactive: 3]; kidney [active: 2, inactive: 1, suspected with abnormal kidney function: 3]; and skin [inactive 1]); [2]Based on the revised Sapporo APS classification criteria; [3]For those fulfilling the revised Sapporo APS classification criteria; [4]For those not fulfilling the revised Sapporo APS classification criteria; [5]Anticoagulantion include warfarin (n: 17), low-molecular-weight-heparin (n: 4), or direct-oral-anticoagulants (n: 2); and [6]Immunosupression includes mycophenolate mofetil, rituximab, or intravenous immunoglobulin.

Supplement Table 2: Antiphospholipid Antibody (aPL) Profile of Persistently aPL-positive Patients (n: 33)

**Total: 33**	**Triple aPL (LA, aCL & aB$_2$GPI) (n:21)**	**Single LA (4) or Double aPL (1) (LA & aCL or aB$_2$GPI) (n:5)**	**Double aPL (aCL & aB$_2$GPI) (n: 5)**	**Single aPL (aB$_2$GPI) (n:2)**
**aCL/aβ$_2$GPI Profile**				
**- IgG +/-M $\geq$ 40U**	20	1	0	1
**- IgM $\geq$ 40U Only**	1	0	5	1
**aPS/PT Positivity**				
**- IgG Only**	7	2	0	0
**- IgG+M**	6	0	0	0
**- IgM Only**	7	1	3	0
**- Negative**	1	2	2	2

LA: lupus anticoagulant; **aCL:** anticardiolipin antibodies; **aβ$_2$GPI**: anti-β$_2$-Glycoprotein-I antibodies**; and aPS/PT:** anti-phosphatidylserine-dependent prothrombin antibody.

Supplement Table 3: Cell-bound Complement Activation Products in Persistently Antiphospholipid Antibody (aPL) Positive Patients without Other Systemic Rheumatic Autoimmune Diseases, Baseline Results Overall and by aPL Profile (for patients with more than one visits and longitudinal samples) (n:20):

#abnormal/# tested or Mean (SD)	**Total (n: 20)**	**Triple aPL (LA, aCL & aB_2GPI) (n:13)***	**Single LA or Double aPL (LA & aCL or aB_2GPI) (n:4)**	**Double aPL (aCL & aB_2GPI) (n: 3)**	**Single aPL (aB_2GPI) (n:0)**
**aPL Details & aCL/aB_2GPI Isotype**	N/A	IgG +/- M: 12<br>IgM Alone: 1	LA Alone: 3	IgM: 3	-
**BC4d Level (MFI)**					
61-100	1/19 (5%)	1/12	0	0	0
101-200	2/19 (11%)	2/12	0	0	0
>200	0	0	0	0	0
Any Positive	3/19 (16%)	3/12	0	0	0
**EC4d Level (MFI)**					
15-30	4/20 (20%)	4/13	0	0	0
31-75 MFI	2/20 (10%)	2/13	0	0	0
>75 MFI	1/20 (5%)	1/13	0	0	0
Any Positive	7/20 (35%)	7/13	0	0	0
**PC4d Level (MFI)**					
10-15 MFI	2/20 (10%)	2/13	0	0	0
16-20 MFI	0	0	0	0	0
>20 MFI	5/20 (25%)	3/13	2/4	0	0
Any Positive	7/20 (35%)	5/13	2/4	0	0

Positive as 61-200 mean fluorescent intensity (MFI), 15-75 MFI, and 10-20 MFI, and **Strongly Positive** above 200 MFI, 75 MFI, and 20 MFI for BC4d, EC4d, and PC4d, respectively. **LA:** lupus anticoagulant; **aCL:** anticardiolipin antibodies; and **$a\beta_2$GPI**: anti-β_2-Glycoprotein-I antibody**.** *One patient with no BC4d result.

Supplement Table 4: Cell-bound Complement Activation Products in Persistently Antiphospholipid Antibody (aPL) Positive Patients without Other Systemic Autoimmune Diseases, Baseline Results Overall and by Clinical Phenotype (<u>for patients with more than one visits and longitudinal samples</u>) (n:20):

#abnormal/# tested or Mean (SD)	**Total (n: 20)**	**MAPS/TP/HA (n:8)***	**TAPS (n: 8)**	**No APS (n: 4)**
**BC4d Level (MFI)**				
61-100	1/19 (5%)	1/7	0	0
101-200	2/19 (11%)	1/7	1/8	0
>200	0	0	0	0
Any Positive	3/19 (16%)	2/7	1/8	0
**EC4d Level (MFI)**				
15-30	4/20 (20%)	1/8	2/8	1/4
31-75 MFI	2/20 (10%)	1/8	1/8	0
>75 MFI	1/20 (5%)	1/8	0	0
Any Positive	7/20 (35%)	3/8	3/8	1/4
**PC4d Level (MFI)**				
10-15 MFI	2/20 (10%)	0	2/8	0
16-20 MFI	0	0	0	0
>20 MFI	5/20 (25%)	2/8	2/8	1/4
Any Positive	7/20 (35%)	2/8	4/8	1/4

Positive as 61-200 mean fluorescent intensity (MFI), 15-75 MFI, and 10-20 MFI, and S**trongly Positive** above 200 MFI, 75 MFI, and 20 MFI for BC4d, EC4d, and PC4d, respectively. **MAPS**: microvascular antiphospholipid syndrome (APS); **TP**: autoimmune thrombocytopenia; **HA**: autoimmune hemolytic anemia; and **TAPS:** thrombotic APS. *One patient with no BC4d result.

Supplement Figure 1: Longitudinal Follow-up of Cell-bound Complement Activation Products (CB-CAPs) in Persistently Antiphospholipid Antibody (aPL) Positive Patients without Other Systemic Autoimmune Diseases (patients only with elevated baseline CB-CAPs [B-lymphocyte - BC4d, erythrocyte - EC4d, or platelet - PC4d] levels are included in the graph)

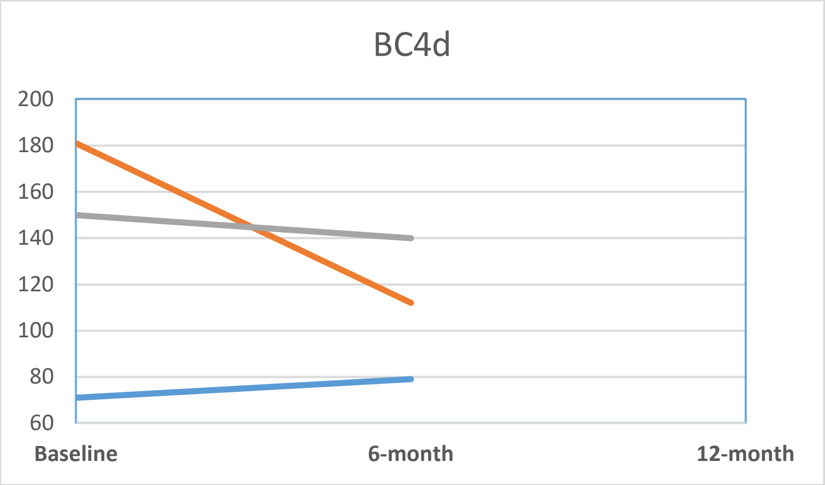

Figure 1A: Based on three patients who had baseline and 6-month **BC4d** results (181 MFI, 150, and 71 *versus* 112, 140, and 79, respectively).

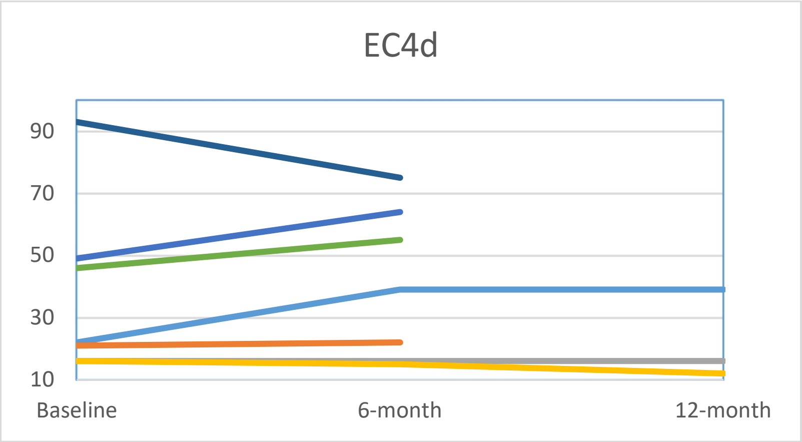

Figure 1B: Based on: a) seven patients who had baseline and 6-month **EC4d** results (93 MFI, 49, 46, 22, 21, 16, and 16 *versus* 75, 64, 55, 39, 22, 16, and 15, respectively); and b) three patients who had baseline and 12-month **EC4d** results (22 MFI, 16, and 16 *versus* 39, 16, and 12, respectively).

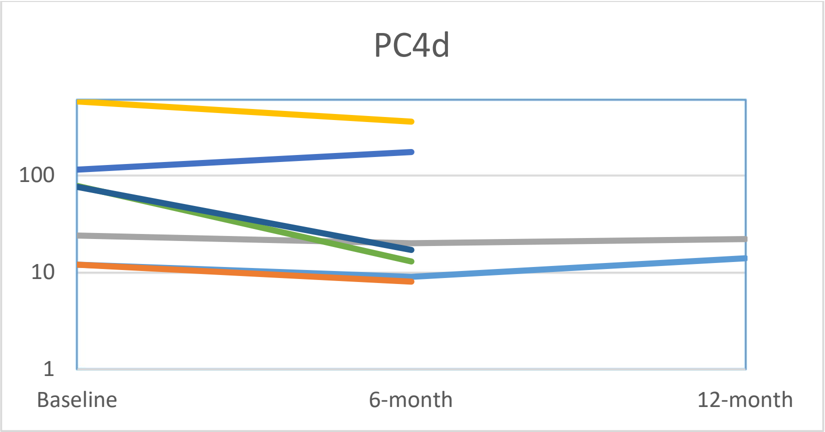

Figure 1C: Based on: a) seven patients who had baseline and 6-month **PC4d** results (579 MFI, 115, 78, 76, 24, 12, and 12 *versus* 358, 174, 13, 17, 20, 9, and 8, respectively); and b) three patients who had baseline and 12-month **PC4d** results (24 MFI, 12, and 12 *versus* 22, 14, and 0, respectively). Data presented on a logarithmic scale given the large range of values.
